# *Blastomyces* Urine Antigen Testing for Active Case Identification During a Blastomycosis Outbreak

**DOI:** 10.3201/eid3203.250973

**Published:** 2026-03

**Authors:** Allyson W. O’Connor, Ian Hennessee, Perri C. Callaway, Marcia L. Stanton, Xiaoming Liang, Ju-Hyeong Park, Ryan LeBouf, Rachel L. Bailey, Rebecca Reik, Mary Grace Stobierski, Michael Snyder, Robert Yin, Mitsuru Toda, Jean Cox-Ganser, Stella E. Hines

**Affiliations:** Centers for Disease Control and Prevention, Morgantown, West Virginia, USA (A.W. O'Connor, P.C. Callaway, M.L. Stanton, X. Liang, J.-H. Park, R. LeBouf, R.L. Bailey, J. Cox-Ganser, S.E. Hines); Centers for Disease Control and Prevention, Atlanta, Georgia, USA (I. Hennessee, P.C. Callaway, M. Toda); Michigan Department of Health and Human Services, Lansing, Michigan, USA (R. Reik, M.G. Stobierski); Public Health Delta & Menominee Counties, Escanaba, Michigan, USA (M. Snyder, R. Yin)

**Keywords:** Blastomycosis, *Blastomyces* spp., fungi, fungal infection, infectious disease outbreaks, exposure assessment, urine antigen test, case identification, United States

## Abstract

*Blastomyces* urine antigen testing is a sensitive blastomycosis diagnostic method, but its utility for active case identification during outbreaks is unknown. We evaluated urine antigen testing for identifying blastomycosis cases during a 2023 outbreak at a Michigan, USA, paper mill and assessed demographic and clinical factors associated with test positivity. Approximately 2 months after the outbreak was recognized, we collected work and health information for 603 employees; 95% (n = 578) underwent urine antigen testing and 9% (n = 52) tested positive, including 25 previously undetected cases. Blastomycosis-like symptoms were associated with test positivity (p<0.001), but 10% of employees with positive results were asymptomatic. Recent hospitalization for blastomycosis was associated with test positivity (p = 0.02) and higher antigen levels. Further research into urine antigen testing is needed clarify its suitability for detecting mild and asymptomatic infections during outbreak investigations. Urine antigen testing had high acceptability among employees and effectively identified additional cases.

Blastomycosis is an environmentally acquired fungal disease caused by *Blastomyces* spp. Pulmonary and systemic manifestations such as cough, chest pain, and fever are the most common (*1*). Blastomycosis is often misdiagnosed, which can lead to inappropriate antibiotic drug use and increased use of healthcare services (*2*). Early detection and treatment can improve patient outcomes, particularly in immunocompromised patients with an elevated risk for severe disease (*3*).

Blastomycosis can be severe; a substantial proportion of cases reported through public health surveillance require hospitalization (*4*,*5*). However, an estimated 50% of infections are asymptomatic (*6*). Detecting asymptomatic infections is necessary for assessing exposure sources during outbreaks (*6*,*7*). Infections may be presymptomatic, and thus hard to detect, given the disease’s long incubation period of 2 weeks to 3 months (*6*,*8*), and reactivation of latent infections can occur after subsequent immunosuppression (*9*).

In 2023, a large outbreak of blastomycosis caused by *Blastomyces gilchristii* occurred among employees at a paper mill in northern Michigan, USA (*10*). A total of 162 cases were identified over the course of the outbreak, with a case prevalence of almost 20% among the mill employees (*11*). As part of a multiagency response, the National Institute for Occupational Safety and Health (NIOSH) led a workplace evaluation and medical survey in late April 2023 that offered *Blastomyces* urine antigen testing to all mill employees and contractors (*11*,*12*). The aim was to identify previously undiagnosed cases among employees and to assess *Blastomyces* exposure to determine potential locations and activities within the mill where exposure might have occurred.

Exposure assessments during blastomycosis outbreaks traditionally evaluate immune responses against the fungus by serologic or skin testing (*6*,*7*,*13*–*15*). However, skin tests are no longer widely available, and most serologic tests have low sensitivity (*16*). Although a newly approved enzyme immunoassay antibody test offers improved sensitivity for serologic testing (*17*), it is unclear how long antibodies remain in blood, making it challenging to distinguish recent infections from historical exposures in endemic areas (*18*). Blood collection poses financial and logistical constraints for large-scale exposure assessments, requiring trained phlebotomists, attention to biosafety concerns, and intensive laboratory processing. Further, participant consent for blood collection during outbreak investigations can be lower than for other specimen types (*19*).

*Blastomyces* urine antigen testing has high sensitivity in symptomatic populations (*16*). Urine samples are easier to collect and process than blood, and urine has generally higher consent rates for sampling. Antigen levels can remain detectable in urine for months after infection (*20*), but antigen levels decline quicker than serum antibodies (*21*). Therefore, positive urine antigen tests might more accurately reflect recent infections, especially when used in endemic areas with potentially elevated background antibody levels among the population. The test also has high specificity, except for substantial cross-reactivity with *Histoplasma* and other fungal pathogens (*22*). The test can have cross-reactivity with other fungal pathogens because it targets galactomannan, a fungal cell wall component common to multiple fungal pathogens. However, cross-reactivity is of less importance in outbreak settings where patients share common exposures to a known fungal pathogen. In this outbreak, 15 cases were confirmed by culture, and whole-genome sequencing of *Blastomyces* isolates from 8 patients revealed strong phylogenetic relatedness (*10*).

We used *Blastomyces* urine antigen testing for the medical survey because of its high sensitivity, higher likelihood of worker participation, and convenience. However, little is known about the test’s effectiveness in identifying new cases during an outbreak. Using the medical survey results, we evaluated the performance of urine antigen testing in actively identifying potential blastomycosis cases without a recent diagnosis or with few or no symptoms and identified demographic and clinical factors associated with *Blastomyces* antigen test positivity.

## Methods

### Data and Population

All mill employees and contractors were invited to participate in NIOSH’s medical survey during April 22–28, 2023, approximately 2 months after the first outbreak cases were recognized. The survey included an interviewer-administered work and health questionnaire and *Blastomyces* urine antigen testing (*12*). We sought written informed consent before participation in the medical survey and antigen testing. Of nearly 1,000 mill employees, 608 completed the survey. Among those who completed the survey, 95% (n = 578) underwent urine testing. We excluded 5 records because of missing or incomplete questionnaire data; 94% (n = 573) employees were included in urine antigen testing analyses. Previous reports describe the outbreak response and clinical characteristics of cases in detail (*10*,*11*). We supplemented survey data with case information collected by the Michigan Department of Health and Human Services (MDHHS) and the local health department through routine surveillance and case interviews. MDHHS relied on a modified version of the national surveillance standard case definition for blastomycosis to classify outbreak-associated blastomycosis cases on the basis of clinical and laboratory criteria (*10*).

### Urine Antigen Testing

We refrigerated urine samples collected during the NIOSH survey and then sent the samples to a commercial laboratory for analysis using the MiraVista quantitative *Blastomyces* antigen enzyme immunoassay test (*22*). At the time of the survey, the test’s quantifiable range for *Blastomyces* antigen was 0.2–14.7 ng/mL, within which antigen levels were reliably quantified. Positive results included any test with detectable antigen levels, including detectable levels that were below the lower limit of quantification (LLOQ) of 0.2 ng/mL and those within or above the quantifiable range. Tests with results below the LLOQ are not considered cases by standard surveillance case definition laboratory criteria, but we counted them as positive results in the modified outbreak case definition. We considered tests with no antigen detected negative. We mailed results to employees and their authorized healthcare providers; positive results were also communicated to employees by phone. Employees with positive results, including those with few or no symptoms, were encouraged to discuss their results with their healthcare providers.

### Questionnaire Data

The interviewer-administered NIOSH questionnaire gathered work and health information from mill employees. We collected information on demographic characteristics, including age and sex, and clinical characteristics, including self-reported symptoms and medical findings, hospitalization and antifungal medication use, and history of potentially immunocompromising health conditions. The questionnaire sought information about symptoms including cough, fever or chills or night sweats, shortness of breath, poor appetite or weight loss, muscle aches or pain, joint or bone pain, and fatigue. We considered employees who did not report any of those symptoms asymptomatic. Medical findings included abnormal lung findings on chest imaging, skin lesions, brain inflammation, abscess, granuloma, other lesions, and bone or joint abnormalities. We collected information on reported symptoms and medical findings that were clinically compatible (*23*) with blastomycosis since October 1, 2022, the earliest date of possible exposure on the basis of available case information. We excluded reported symptoms and medical findings that occurred within 2 weeks of a self-reported COVID-19, influenza, or respiratory syncytial virus illness. We also collected self-reported information on potential immunocompromising conditions, including diabetes, autoimmune diseases, taking immunosuppressive medication, and history of an organ or stem cell transplant.

We classified employees as having a recent diagnosis of blastomycosis during the outbreak if they were listed as having a confirmed or probable case of blastomycosis on the MDHHS case list as of April 21, 2023, or if they self-reported a healthcare provider diagnosis since October 1, 2022, on the NIOSH questionnaire. We asked employees with recent blastomycosis diagnoses if they were hospitalized for their recent illness and if they had initiated antifungal drug treatment.

### Data Analysis

We summarized worker characteristics, including recent blastomycosis diagnoses during the outbreak, by urine antigen test positivity. We then assessed associations between clinical characteristics and test positivity among all participants who underwent testing and among employees with a recent blastomycosis diagnosis during the outbreak. We also examined clinical characteristics by antigen levels (below the LLOQ vs. within or above the quantifiable range) among employees with positive tests. We evaluated bivariate associations between worker or clinical characteristics and test positivity by using Wilcoxon rank sum tests for continuous variables (to compare the medians) and Pearson’s χ^2^ or Fisher exact tests for categorical variables, with 2-sided p values of 0.05 indicating statistical significance. We did not assess statistical associations for antigen levels because of the small numbers in this analysis.

We conducted statistical analyses by using SAS software version 9.4 (SAS Institute, Inc.). This activity was reviewed by the Centers for Disease Control and Prevention, was deemed not research, and was consistent with applicable federal law and Centers for Disease Control and Prevention policy.

## Results

Among 573 employees who underwent *Blastomyces* urine antigen testing during the medical survey period, the median age was 47 years (interquartile range [IQR] 38–53 years); 82% were male and 18% female, and 12% had a recent diagnosis of blastomycosis during the outbreak (Table 1). Of the 573 employees, 52 (9%) received positive *Blastomyces* antigen test results; there were no significant differences in test positivity by age (p = 0.42) or sex (p = 0.61). A recent diagnosis of blastomycosis during the outbreak was associated with test positivity (p<0.001); of 71 participants with a recent diagnosis, 27 (38%) tested positive. In addition, 25 (48%) of 52 employees who tested positive were previously undiagnosed.

**Table 1:** Characteristics of employees by UAT result from a medical survey conducted in a study of *Blastomyces* urine antigen testing for active case identification during a blastomycosis outbreak, United States*

Characteristic	Total, n = 573	Positive UAT, n = 52	Negative UAT, n = 521	p value†
Median age, years (IQR)	47 (38–53)	46 (38–53)	47 (38–54)	0.42
Sex				
M	470 (82)	44 (85)	426 (82)	0.61
F	103 (18)	8 (15)	95 (18)	
Recent blastomycosis diagnosis‡				
Yes	71 (12)	27 (52)	44 (8)	<0.001
No	502 (88)	25 (48)	477 (92)	

*Values are no. (%) except as indicated. Numbers might not reach column total because of missing responses. UAT, urine antigen test; IQR, interquartile range. †Wilcoxon rank sum tests for continuous variables and Pearson χ^2^ tests or Fisher exact tests for categorical variables were used to test differences by urine antigen test result. ‡Recent diagnosis of blastomycosis included employees the Michigan Department of Health and Human Services identified as having confirmed or probable blastomycosis during the paper mill outbreak as of April 21, 2023, or self-reported healthcare provider-diagnosed blastomycosis on the medical survey questionnaire.

Among the 573 survey participants, 67% reported symptoms, and the number of symptoms differed significantly by test positivity (p<0.001). Among 52 employees with positive tests, 90% reported >1 symptom, compared with 65% of 521 employees who tested negative; 10% of test-positive employees were asymptomatic (Table 2). Four of the 5 asymptomatic cases were from previously undiagnosed employees (data not shown). Reporting any medical findings was also more common among test-positive employees (46%) than test-negative employees (13%; p<0.001). All individual blastomycosis-associated symptoms (e.g., cough, fever, shortness of breath) and medical findings (e.g., abnormal lung imaging) were also more common among test-positive employees (p<0.05) (Appendix Table 1, https://wwwnc.cdc.gov/EID/article/32/3/25-0973-App1.pdf). Immunocompromising conditions were not associated with test positivity for employees in the survey (p = 0.58).

**Table 2:** Clinical factors by UAT result among employees from a medical survey conducted in a study of *Blastomyces* urine antigen testing for active case identification during a blastomycosis outbreak, United States*

Clinical factor	All employees					Employees with recent blastomycosis diagnosis‡			
	Total, n = 573	Positive UAT, n = 52	Negative UAT, n = 521	p value†		Total, n = 71	Positive UAT, n = 27	Negative UAT, n = 44	p value†
No. symptoms potentially related to blastomycosis									
0	184 (32)	5 (10)	179 (34)	<0.001		4 (6)	1 (4)	3 (7)	0.75
1	156 (27)	5 (10)	151 (29)			5 (7)	1 (4)	4 (9)	
>2	231 (40)	42 (81)	189 (36)			62 (87)	25 (93)	37 (84)	
No. medical findings potentially related to blastomycosis									
0	479 (84)	28 (54)	451 (87)	<0.001		16 (23)	6 (22)	10 (23)	0.96
>1	92 (16)	24 (46)	68 (13)			55 (77)	21 (78)	34 (77)	
Days from symptom onset to test, median (IQR)§	NA	NA	NA	NA		48 (39–69)	47 (31–70)	52 (40–69)	0.32
Potentially immunocompromised¶									
Yes	64 (11)	7 (13)	57 (11)	0.58		13 (18)	6 (22)	7 (16)	0.54
No	509 (89)	45 (87)	464 (89)			58 (82)	21 (78)	37 (84)	
Pneumonia diagnosis									
Yes	26 (5)	8 (15)	18 (3)	0.001		17 (24)	7 (26)	10 (23)	0.76
No	543 (95)	44 (85)	499 (97)			54 (76)	20 (74)	34 (77)	
Hospitalized for blastomycosis§									
Yes	NA	NA	NA			11 (16)	8 (31)	3 (7)	0.02
No	NA	NA	NA			58 (84)	18 (69)	40 (93)	
Antifungal drugs initiated§									
Yes	NA	NA	NA			65 (93)	25 (96)	40 (91)	0.64
No	NA	NA	NA			5 (7)	1 (4)	4 (9)	
Days from antifungal drug initiation to antigen test, median (IQR)§	NA	NA	NA			31 (18–41)	29 (13–41)	33 (22–40)	0.36

*Values are no. (%) except as indicated. Numbers might not reach column total because of missing responses. UAT, urine antigen test; IQR, interquartile range; NA, not applicable. †Wilcoxon rank sum tests for continuous variables and Pearson χ^2^ tests or Fisher exact tests for categorical variables were used to test differences by urine antigen test result. ‡Recent diagnosis of blastomycosis included employees the Michigan Department of Health and Human Services identified as having confirmed or probable blastomycosis during the paper mill outbreak as of April 21, 2023, or self-reported healthcare provider-diagnosed blastomycosis on the NIOSH medical survey questionnaire. §Data were only collected for employees with a recent diagnosis of blastomycosis (n = 71) during the outbreak and thus not available for all employees. ¶Potentially immunocompromised was defined as reporting diabetes, an autoimmune disease, taking immunosuppressive medication, or an organ transplant.

Among 71 employees with a recent diagnosis of blastomycosis, 93% reported >1 symptom, and 77% reported >1 medical finding (Table 2). Unlike in the full survey population, the number of symptoms (p = 0.75) or medical findings (p = 0.96) did not differ significantly by test positivity among those employees. There was no statistical difference in the median time from symptom onset to the survey urine antigen testing between test-positive employees (47 days, IQR 31–70 days) and test-negative employees (52 days, IQR 40–69 days; p = 0.32). Sixty-five (93%) employees initiated antifungal treatment a median of 31 (IQR 18–41) days before survey testing. Neither treatment initiation (p = 0.64) nor time from initiation to testing during the survey (p = 0.36) were associated with test positivity. Recent hospitalization for blastomycosis was associated with test positivity (p = 0.02); among 27 recently diagnosed employees with positive tests, 31% reported hospitalization, compared with 7% of 44 recently diagnosed employees who tested negative.

Among the 52 employees with positive tests, 17 (33%) employees had *Blastomyces* antigen results below the LLOQ (<0.2 ng/mL) and 35 (67%) employees had results within or above the quantifiable range (0.2–14.7 ng/mL) (Table 3). Three (18%) of 17 employees with results below the LLOQ reported no symptoms, and another 3 (18%) only reported 1 symptom. Among 35 employees with results within or above the quantifiable range, 2 (6%) were asymptomatic and another 2 (6%) only reported 1 symptom. For test-positive employees reporting symptoms, median time from symptom onset to testing was similar for employees with positive tests below the LLOQ (49 days, IQR 37–70 days) and within or above the quantifiable range (45 days, IQR 23–70 days). Of the 17 employees with results below the LLOQ, 8 employees reported having initiated antifungal drug treatment, whereas the remaining 9 were not asked about treatment because they had no prior blastomycosis diagnosis. The 8 test-positive employees with recent blastomycosis diagnoses who reported recent hospitalization all had test results within or above the quantifiable range, representing 28% of employees with results within or above the quantifiable range. None of the 17 employees with results below the LLOQ reported recent hospitalization.

**Table 3:** Clinical factors by UAT levels for employees with positive tests from the medical survey conducted in a study of *Blastomyces* urine antigen testing for active case identification during a blastomycosis outbreak, United States*

Clinical factor	Positive UAT result, n = 52	
	Below LLOQ, n = 17	Within or above quantifiable range, n = 35
No. symptoms potentially related to blastomycosis		
0	3 (18)	2 (6)
1	3 (18)	2 (6)
>2	11 (65)	31 (89)
No. medical findings potentially related to blastomycosis		
0	10 (59)	18 (51)
>1	7 (41)	17 (49)
Days from symptom onset to test, median (IQR)†	49 (37–70)	45 (23–70)
Potentially immunocompromised‡		
Yes	2 (12)	5 (14)
No	15 (88)	30 (86)
Had a recent blastomycosis diagnosis		
Yes	8 (47)	19 (54)
No	9 (53)	16 (46)
Pneumonia diagnosis		
Yes	2 (12)	6 (17)
No	15 (88)	29 (83)
Hospitalized for blastomycosis†		
Yes	0	8 (28)
No	17 (100)	21 (72)
Antifungal drug initiated†		
Yes	8 (100)	17 (94)
No	0	1 (6)
Days from antifungal drug initiation to antigen test, median (IQR)†	25 (15–38)	31 (13–43)

*Values are no. (%) except as indicated. Numbers might not reach column total because of missing responses. LLOQ was 0.2 ng/mL, quantifiable range was 0.2–14.7 ng/mL. UAT, urine antigen test; LLOQ, lower limit of quantification. †Data were only collected for employees with a recent diagnosis of blastomycosis during the outbreak (n = 71) and thus not available for all employees. ‡Potentially immunocompromised was defined as reporting diabetes, an autoimmune disease, taking immunosuppressive medication, or an organ transplant.

## Discussion

Our investigation used *Blastomyces* urine antigen testing to actively identify unknown or asymptomatic cases during a blastomycosis outbreak. The test successfully identified additional cases; nearly half the positive results came from employees without recent blastomycosis diagnoses during the outbreak. The 25 new cases identified through urine antigen testing, including cases in 4 employees who were asymptomatic, accounted for 15% of 162 cases ultimately identified during the outbreak. Urine antigen testing increased case identification beyond routine surveillance and contributed to a comprehensive outbreak investigation (*11*,*12*). Test acceptability was high, with 95% of employees who completed the survey questionnaire also consenting to testing.

Although urine antigen testing identified undiagnosed symptomatic cases, it appeared less effective for identifying asymptomatic infections. *Blastomyces* urine antigen tests were not originally developed to detect *Blastomyces* infection in asymptomatic persons, and our findings suggest their utility in this context might be limited. Only 10% of employees with positive urine antigen tests during this evaluation were asymptomatic, whereas several prior investigations that used testing for specific immune responses estimated that ≈50% of *Blastomyces* infections are asymptomatic (*6*,*24*–*26*).

The lower-than-expected proportion of asymptomatic employees with positive tests suggests that urine antigen testing might be less sensitive than immunological testing for identifying asymptomatic or mild infections. Previous studies reported lower sensitivity and lower antigen levels among patients with mild disease compared with patients with more severe disease (*10*,*27*–*29*). Those data align with our findings that fewer employees reporting no symptoms or a single symptom had positive tests and antigen levels within or above the quantifiable range compared with those who had >2 symptoms. In addition, employees who were hospitalized for blastomycosis during the outbreak, an indicator of more severe disease, more frequently had test results within or above the quantifiable range compared with those who were not hospitalized.

Several factors related to the timing of testing could also explain the lower-than-expected rate of asymptomatic infections identified through urine antigen tests. *Blastomyces* exposures likely occurred over several months during this outbreak, peaking in January or February 2023 (*11*). For some employees, antigen levels might have declined by the time urine antigen testing was offered in late April; ≈80% of confirmed and probable cases reported to MDHHS had initial positive urine antigen tests (*10*), whereas only 38% of employees with recent diagnoses tested positive during this survey. However, we did not have information on what type of testing employees who self-reported recent blastomycosis diagnoses received, which limited our ability to directly assess declining antigen levels among this group. Antigen levels might have declined particularly quickly in employees with mild or asymptomatic infections, as antigen levels are positively associated with disease severity, and declining antigen levels correlate with clinical improvement (*20*,*30*). In contrast, all hospitalized employees with recent blastomycosis diagnoses who tested positive in the survey had results within or above the quantifiable range at the time of the survey. It is also unknown whether sustained exposure over several months affects progression or detectability of antigenuria compared with shorter-term exposure.

Antigen levels might have also declined more rapidly in employees taking antifungal drugs; 93% of employees with recent diagnoses during the outbreak had received antifungal drugs. A previous study reported initial spikes in antigen levels ≈11 days after antifungal drug treatment and declining antigen levels in the following months, although the median time to first negative result was 200 days (*20*). We did not find an association between antigen test positivity during the survey and antifungal drug treatment or days from antifungal drug treatment initiation to testing, but we were likely limited in the power to detect any associations, because only 5 employees with recent diagnoses had not received antifungal drugs. Although no employees reported having a prior blastomycosis diagnosis before the outbreak, prior exposure or undiagnosed infections could have conferred immunity and influenced antigen levels in this population.

An alternative explanation to the low observed rate of asymptomatic infections could be that we overestimated the number of symptomatic employees. We collected symptom information over a 7-month period on the questionnaire. Although we excluded symptoms related to self-reported respiratory infections, such as COVID-19 or influenza, we might have attributed reported symptoms to blastomycosis when they were because of another illness. Second, this outbreak occurred in a relatively healthy cohort of employees, which might limit the generalizability of our findings to other populations. Although we did not detect an association between test positivity and potential immunocompromise, our cohort included few employees with immunocompromising conditions; however, we did not have complete information for conditions such as HIV or malignancy, which are associated with increased blastomycosis risk (*3*). Last, heightened awareness of the outbreak among employees at the mill and the local healthcare community likely led to early identification of more cases, including less severe ones, than standard public health surveillance (*10*). Early care seeking and treatment could have reduced the overall severity of cases in this outbreak and potentially influenced urine antigen test positivity rates.

In conclusion, our findings suggest urine antigen testing might be less effective for detecting asymptomatic infections during investigations where exposure assessment is the primary goal. In such situations, serologic testing might be a better choice, but longitudinal studies comparing methods for detecting asymptomatic and mild infections over several months after exposure are needed to help clarify this consideration. Despite being potentially less effective for asymptomatic testing, our findings do indicate that *Blastomyces* urine antigen testing is a viable option for actively identifying cases during future blastomycosis outbreaks, in part because of its logistical ease and high acceptability compared with methods that rely on blood collection. Urine antigen testing might be particularly advantageous when used during outbreaks within a large exposed population (*11*). 

Appendix Additional information about *Blastomyces* urine antigen testing for active case identification during a blastomycosis outbreak.
