# Supplementary material for: Blastomyces Urine Antigen Testing for Active Case Identification During a Blastomycosis Outbreak
Source: Emerg Infect Dis. 2026 Mar;32(3):360–7. doi: 10.3201/eid3203.250973 (PMC13016013; doi:10.3201/eid3203.250973)
Supplement: Appendix — Additional information about Blastomyces urine antigen testing for active case identification during a blastomycosis outbreak. [file 25-0973-Techapp-s1.pdf]

Article DOI: https://doi.org/10.3201/eid3203.250973

EID cannot ensure accessibility for supplementary materials supplied by authors. Readers who have difficulty accessing supplementary content should contact the authors for assistance.

Blastomyces Urine Antigen Testing for Active Case Identification During A Blastomycosis Outbreak

Appendix

Appendix Table. Health symptoms and medical findings for workers who underwent urine antigen testing (UAT)

	All Workers			
Health symptom or medical finding, No. (%)	**Total** (n=573)	**UAT Positive** (n=52)	**UAT Negative** (n=521)	P-value*
Cough	327 (57)	40 (77)	287 (55)	0.003
Fever or chills or night sweats	147 (26)	33 (63)	114 (22)	<.0001
Shortness of breath, or working harder than normal to breathe	154 (27)	30 (58)	124 (24)	<.0001
Poor appetite or unexpected weight loss	37 (6)	13 (25)	24 (5)	<.0001
Muscle aches or pain	109 (19)	24 (46)	85 (16)	<.0001
Joint pain or bone pain	78 (14)	19 (37)	59 (11)	<.0001
Fatigue or extreme tiredness	149 (26)	29 (56)	120 (23)	<.0001
Abnormal lung findings on chest imaging, like on an x-ray or CT scan	68 (12)	20 (38)	48 (9)	<.0001
Skin lesions with no known cause, such as raised bumps, blisters, or ulcers	23 (4)	5 (10)	18 (3)	0.049

Note: Numbers (%) are the number of workers who reported the symptom versus those who did not report the symptom out of all workers in the survey. Symptoms and medical findings were reported since October 1, 2022, excluding those which occurred within two weeks of a self-reported COVID-19, influenza, or respiratory syncytial virus illness.
